# Segmentation of liver, its vessels and lesions from CT images for surgical planning

**DOI:** 10.1186/1475-925X-10-30

**Published:** 2011-04-20

**Authors:** Dário AB Oliveira, Raul Q Feitosa, Mauro M Correia

**Affiliations:** 1 Electrical Engineering Department, Pontifical Catholic University of Rio de Janeiro, Marquês de São Vicente 225, Rio de Janeiro, RJ, Brasil; 2 Department of Surgery, School of Medicine, Unigranrio and National Cancer Institute-INCA, Brazil

## Abstract

**Background:**

Cancer treatments are complex and involve different actions, which include many times a surgical procedure. Medical imaging provides important information for surgical planning, and it usually demands a proper segmentation, i.e., the identification of meaningful objects, such as organs and lesions. This study proposes a methodology to segment the liver, its vessels and nodules from computer tomography images for surgical planning.

**Methods:**

The proposed methodology consists of four steps executed sequentially: segmentation of liver, segmentation of vessels and nodules, identification of hepatic and portal veins, and segmentation of Couinaud anatomical segments. Firstly, the liver is segmented by a method based on a deformable model implemented through level sets, of which parameters are adjusted by using a supervised optimization procedure. Secondly, a mixture model is used to segment nodules and vessels through a region growing process. Then, the identification of hepatic and portal veins is performed using liver anatomical knowledge and a vein tracking algorithm. Finally, the Couinaud anatomical segments are identified according to the anatomical liver model proposed by Couinaud.

**Results:**

Experiments were conducted using data and metrics brought from the liver segmentation competition held in the Sliver07 conference. A subset of five exams was used for estimation of segmentation parameter values, while 15 exams were used for evaluation. The method attained a good performance in 17 of the 20 exams, being ranked as the 6^th ^best semi-automatic method when comparing to the methods described on the Sliver07 website (2008). It attained visual consistent results for nodules and veins segmentation, and we compiled the results, showing the best, worst, and mean results for all dataset.

**Conclusions:**

The method for liver segmentation performed well, according to the results of the numerical evaluation implemented, and the segmentation of liver internal structures were consistent with the anatomy of the liver, as confirmed by a specialist. The analysis provided evidences that the method to segment the liver may be applied to segment other organs, especially to those whose distribution of voxel intensities is nearly Gaussian shaped.

## Background

In medical image analysis, image-guided surgery and organ visualization, segmentation is a crucial step. The segmentation process is particularly arduous in abdominal computer tomography (CT) images because different organs lie within overlapping intensity ranges and are often near to each other anatomically. Therefore, usually it is not possible to define accurately the boundaries of organs, their vessels and lesions using simple threshold based segmentation. On the other hand more complex algorithms involve comparatively many parameters of which adjustment is not a simple issue.

Numerous techniques have been proposed in the literature for extraction of organ contours in abdominal CT scans. They can be roughly divided in two main groups: model driven and data driven approaches [1].

Model driven techniques (e.g. [2,3]) use pre-defined models to segment the meaningful objects in the images being analyzed. In this kind of technique a model describing the organ to be segmented is defined in terms of object characteristics such as position, texture and spatial relation to other objects, and the algorithm searches the images for instances that fit the given model.

Data driven techniques (e.g. [4,5]) try to emulate the human capacity of identifying objects using some similarity information present on image data, automatically detecting and classifying objects and features in images. Many of them use traditional techniques such as region growing and thresholds, combined with some prior knowledge about the object being analyzed.

Level set methods [6] are model driven methods that rely on partial differential equations to model deforming isosurfaces. These methods have been used successfully in medical image processing but usually require human intervention to set an initial solution and indicate explicitly when the model should stop expanding. Moreover, semi automatic level set based methods involve a time consuming trial and error procedure for optimum parameter tuning.

The parameters in the implementation of traditional level sets are related to the curves mean curvature, propagation rate and advection of the curve to certain characteristics of the image. The manual definition of these values on level set methods is a complex task, because their relation with the final result is unclear and there is no guarantee that the optimal set of values will be found. Therefore, there is a demand for methods to define such parameters automatically.

Some works approaching liver segmentation using level set based methods are found on the literature. In [7] a level set method without edges was proposed to segment the liver, using the Chan-Vese methodology ([8]). In [9] an active model based on level sets was proposed to segment the liver, using a multi-resolution concept to reduce processing time. In both works, in spite of achieving good results, the parameters were not automatically defined, and the segmentation did not included liver vessels and lesions.

In this work we propose a complete methodology to segment the liver ([10-12]) and its internal structures, such as vessels ([11]) and nodules, using level sets, stochastic optimization, and Gaussian mixture model. It also proposes a methodology to separate the liver into segments according to the Couinaud [13] anatomical model.

The subsequent text is organized in the following way. First, the liver anatomy is briefly described. The theoretical fundamentals of level sets and the optimization algorithm used are then presented. Next, we present the proposed liver segmentation method in details and the parameter estimation procedure. Then, the segmentation of vessels and nodules, the identification of portal and hepatic veins and the segmentation of Couinaud regions are described. Last, we report the results of the corresponding experimental performance evaluation, and present the main conclusions.

### Liver Anatomy

The liver is a vital and complex gland, with a wide range of functions, including detoxification, protein synthesis, and production of biochemicals necessary for digestion. It has two distinct descriptions, according to its morphological and functional aspects. Morphologically the liver presents 4 lobes: left, right, caudate and quadrate. Functionally the liver present a different anatomy, and one of the proposed ones is the Couinaud model, which is used within this work.

The functional anatomy model of Couinaud proposes the division of the liver into eight different regions according to the portal and hepatic veins positions:

Segment I - caudate/Spiegel lobe

Segment II - left posterolateral

Segment III - left anterolateral

Segment IVa - left superomedial

Segment IVb - left inferomedial

Segment V - right anteroinferior

Segment VI - right posteroinferior

Segment VII - right posterosuperior

Segment VIII - right anterosuperior

**Figure 1:**
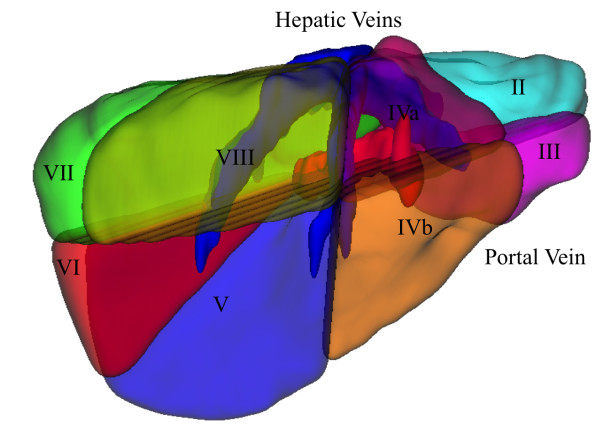
**Liver Functional Anatomy: segments of Couinaud**. Hepatic veins and portal vein define the segments, except segment I, which is drained by cava vein.

As illustrated in figure 1, each segment has its limits defined by the hepatic veins and the portal vein, except segment I, which is drained by the cava vein.

### Level Sets

The level set methods theory was originally developed by Sethian and Osher [6,14] for analyzing and computing moving fronts in a variety of different settings. These methods were firstly introduced in medical imaging by Malladi et al. [15].

The level set methodology relies on a numerical technique that uses level set function to model and compute continuous deformable models [16] using an implicit representation. It has been applied to different areas, such as computer graphics, computer vision and image processing. In image processing it was successfully applied to segment structures with boundaries not clearly defined, that usually cannot be segmented using simple data driven methods, based on thresholds and line extraction. The level set approach takes into account other properties such as mean curvature to model the target object, in such a way that it is possible to avoid undesirable leaks toward neighbouring structures, preserving the boundaries of the segmented object even when they are not clearly defined through the entire object.

The main idea of level sets is to embed a deformable model in a *d+1 *dimensional space, in order to segment iteratively an object in a *d *dimensional space. The main advantage of level sets is that it allows changes of surface topology implicitly. As it embeds the evolving surface, also called interface, in a higher dimensional function, the interface can split into several connected components or merge from different connected components naturally. It occurs because the embedding level set function, which is used to deform the surface, remains continuous.

Considering *ψ*(*x,t*) the level set function, *x *the position vector and *t *the time step of the level set evolution, the evolving surface is represented as the zero level set of *ψ*(*x,t*) *= 0*. This time step controls the evolution of the interface in time. The interface *ψ*(*x,t+1*) *= 0 *is computed taking into account the interface of the previous time step *ψ*(*x,t*) *= 0*, using partial differential equations. The segmentation procedure ends when the RMS difference between *ψ*(*x,t*) *= 0 *and *ψ*(*x,t-1*) *= 0 *is less than a pre-defined minimum RMS value.

The level set function is usually a smooth well behaved function, for numerical stability reasons during partial differential equations computation. This work uses the signed distance function that computes the distance of each voxel to its closest voxel in the interface. This distance is negative inside the interface, and positive outside, in such a way that the position of a given point in relation to the interface can be easily determined by looking at the sign of the level set function in that point.

Using an initial surface *S*_*0*_, which is consequently *ψ*(*x,0*) *= 0*, the level set function evolves under the control of the differential equation 1, that defines the displacement of the interface in a time step.(1)

Note that the gradient *Δψ *(or its module) of *ψ*(*x,t*) appears in all terms of the equation. As *ψ*(*x,t*) was defined as the signed distance function, the gradient of *ψ*(*x,t*) points from inside to outside considering the interface *ψ*(*x,t*) *= 0*, and *|Δψ| = 1*, by definition. It defines, together with other parameters, the direction and magnitude of interface evolution.

The functions *A, P *and *Z *are usually calculated from the input image. *A *is a vector field responsible for the advection term. It attracts the evolving surface to determinate features, usually related to boundaries of objects, and pre-defined barriers. It is weighted by the constant advection weight *α*, and multiplied by the gradient of *ψ*(*x,t*).

*P *is a propagation image, also called speed image, which controls how the level sets propagates through the coordinate space. This image normally has high values in regions where the interface can expand quickly, and values close to zero in regions where it should move slowly or stop (normally close to important features). It is weighted by the constant propagation weight *β *and multiplied by the module of the gradient of *ψ(x,t)*.

*К *is the mean curvature of the interface, and is defined as the divergence of the normal to the interface. It is usually calculated using first and second derivatives of the interface, based on finite differences. In this way, *К > 0 *for convex regions, and *К < 0 *for concave regions. *Z *is a spatial modifier for the mean curvature *К*, and modifies the value of *К *in a determinate spatial position. In this work *P *was defined in such a way that the curvature has less importance close to important features. It is weighted by the constant mean curvature weight *γ *and multiplied by the module of the gradient of *ψ*(*x,t*).

The basic level set model has these three terms but a segmentation algorithm based on level sets may add new different terms, or omit one or more terms, depending on the problem complexity.

### Supervised adaptation of segmentation parameters

Many of the parameters mentioned until now must be properly adjusted for the level set method to produce accurate results. The determination of appropriate parameter values usually cannot be done using heuristics mainly due to the complexity of the target application. It is generally also not possible to determine the optimum values using non-linear optimization methods for parameter adjustment, due to multiple local minima.

A number of automatic methods have been proposed to optimize parameters in such cases. They can be grouped into two categories: supervised and unsupervised [17]. Unsupervised quality assessment methods, e.g., [18] and [19], rely on statistics computed from the image being segmented and the segmentation itself. Supervised methods try to avoid subjectivity through the use of reference segments, which represent an ideal segmentation. Quality is assessed by measuring the deviation of the segmentation outcome from the references.

Stochastic approaches for optimization are options to consider when calculus-based optimization methods cannot be applied, such as in our methodology. Although stochastic methods are computationally intensive and do not guarantee that the optimum will be reached, they are able to work with virtually any objective function.

In the present work we used a genetic algorithm (GA), which is a stochastic technique inspired by the evolution theory and widely applied in many optimization problems [20].

GA methods aim at automatically adjusting segmentation parameters, taking into consideration a set of reference segments, delineated by an interpreter. The basic idea is to search the parameter space for a set of values that optimizes a given fitness function, which expresses numerically the discrepancy among the automatically generated objects and the references. For details refer to [21,22].

It is worth emphasizing that the model described hitherto is not bound to any particular parameter optimization technique. There may be optimization approaches other than GA (e.g. simulated annealing) that could be more efficient for this particular problem. An evaluation of possible optimization techniques that could be used to get the best parameters goes beyond the scope of this paper, but is an interesting issue to be pursued in the continuation of this research. Nevertheless, the results reported attest that our method may achieve good results using a GA for parameter estimation and some approaches for different image segmentation methods were already published (e.g. [23]) with encouraging results.

## Methods

The methodology consists of four steps executed sequentially. Firstly the liver is segmented using level sets with optimal parameters computed by a GA. The level set segmentation uses an initial user-defined liver segment in one slice, and then segments the liver through all other slices, using a Gaussian fit to define the speed image where the level sets propagates. The initial solution at each slice is defined as the region previously segmented on an adjacent slice.

Secondly, the vessels and nodules are segmented using a Gaussian mixture model. In this work only the nodules that appear darker than liver parenchyma, the adipose ones, are contemplated. Thus, the model consists in discriminating Gaussians in a mixture model considering that the vessels appear brighter than the liver parenchyma and the nodules appear darker. Then a region growing method using the information of each Gaussian implements the segmentation.

Thirdly, the vessels are classified into portal vein or hepatic vein using a simple vein tracking method, and finally, a geometrical approach is applied to split the liver into different regions of Couinaud, using the anatomy of these two defined veins.

These steps are described in further details in the following sections.

### Liver Segmentation

The proposed method relies on two hypotheses: the liver parenchyma is roughly homogeneous, and liver veins are mainly inside the liver, as well as liver nodules. The impact of these heuristics on cases where peripheral nodules and veins are present is discussed in details in the section on experimental results.

Firstly an initial solution is defined manually in just one slice. This initial solution is expected to contain great part of the liver, but does not need to be accurate, as it will be later deformed too. Then an iterative process takes place, both upwards and downwards through the whole exam, in which the liver is segmented at each slice sequentially. In this process the initial solution of a new slice to be processed is the previously segmented result of the adjacent slice, which is then deformed towards the liver boundaries using the approach based on level sets, with some of the parameters being adjusted at each slice.

In this work, the advection term was not used, because it is closely related to boundary features and the boundaries of the liver cannot be robustly distinguished from its anatomical neighbouring structures.

We assume that the liver parenchyma is roughly homogeneous and it is possible to define two thresholds *T*_*L *_and *T*_*H *_that define a gray level range where most of parenchyma voxels lie on.

**Figure 2:**
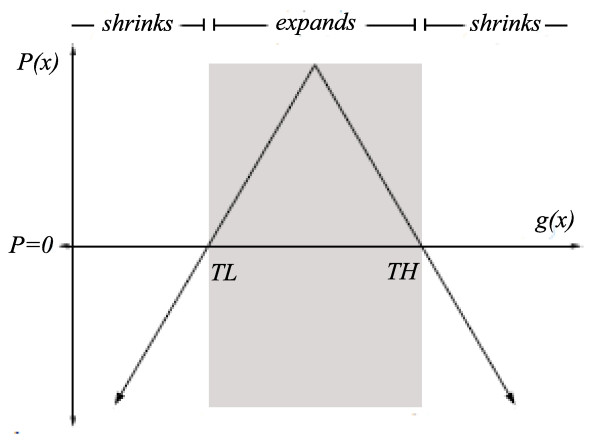
***P(x) *definition**. The speed image is defined by a function based on two automatically defined thresholds *T*_*L *_and *T*_*H *_(*T*_*L*_*<T*_*H*_) and the input image *g(x)*

To define the propagation term one must define the speed image and the propagation weight *β*. We define the speed image as a function parameterized by two thresholds *T*_*L *_and *T*_*H *_(*T*_*L*_*<T*_*H*_) and by the input image *g*(*x*). This function is expressed in equation 2, and depicted in Figure 2:(2)

The propagation *P*(*x*) in the preceding equation is positive when the pixel intensity lies inside the range [*T*_*L*_*,T*_*H*_] and negative when it is outside the range. It implies that the surface expands in regions where pixel values are inside the range, and shrinks otherwise. It can be further observed in equation 2 that the surface evolving speed decreases or even becomes zero, in regions where pixel values are close to *T*_*L *_and *T*_*H*_. In this sense, considering that *T*_*L *_and *T*_*H *_are values close to the ones observed on the liver boundaries, the surface evolution stops when it achieves the liver boundaries.

Clearly, the choice of proper values for *T*_*L *_and *T*_*H *_is crucial for good accuracy. We propose a simple algorithm to set up *T*_*L *_and *T*_*H*_, relying on the assumption that the voxel intensities inside the liver follow a Gaussian shaped distribution.

**Figure 3:**
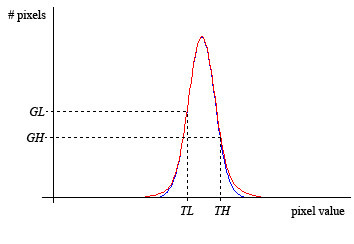
**Gaussian thresholds estimation**. The two thresholds *T*_*L *_and *T*_*H *_are defined as the values for which the Gaussian returns two pre-defined values, respectively *G*_*L *_and *G*_*H*_

The intensity histogram of the region inside the given initial solution at the respective iteration step is computed. Then a Gaussian function is fitted to the histogram, using the well known Levenberg-Marquadt non-linear minimization estimator [24,25], and the two thresholds *T*_*L *_and *T*_*H *_are defined as the values for which the Gaussian returns two pre-defined values, respectively *G*_*L *_and *G*_*H*_, as depicted in Figure 3. In this model, the grey level range [*T*_*L*_*,T*_*H*_] is meant to represent the liver parenchyma. The values of *G*_*L *_and *G*_*H *_are parameters that must be properly defined during the adaptation of parameters but once estimated, they are fixed, while *T*_*L *_and *T*_*H *_vary within each exam. Moreover, it is important to emphasize that the definition of rigid *T*_*H *_and *T*_*L *_values would lead one to face problems with the differences of intensities found in different exams. This formulation using Gaussian fit computed values, provides robustness to the method against this problem.

The mean curvature term is defined by the spatial modifier *Z*, the mean curvature *К *and the weight *γ*. The spatial modifier *Z *was defined like *P*, in such a way that the importance of the mean curvature *К *decreases close to the boundaries. This avoids that boundary details are missed by smoothing too much the surface on boundaries of the segmented object.

Once the values for *G*_*L*_, *G*_*H*_, *β *and *γ *are selected, the level set process can start at a given slice, deforming the initial solution towards the liver boundaries until the convergence criterion is achieved (minimum RMS).

The level set segmentation procedure confine the voxels inside the liver parenchyma, since their intensities meet the estimated Gaussian distribution, but nodules and veins, which normally appear respectively as darker and brighter regions are not captured. This problem is partially eliminated by the use of a 'fill-holes' morphologic algorithm [26], that merges to the result of the preceding step veins and nodules that are totally inside the liver. However the problem persists when a nodule or vein is at the periphery, when they usually do not appear in the final result.

This algorithm is applied sequentially for each slice in the exam, and ends when it achieves the first and last slices, or when an initial segment is vanished by the level set algorithm in a given slice, indicating a limit of the liver.

### Parameter Estimation

#### Processing Scheme

In the devised GA each individual consists of a set of segmentation parameter values; each parameter is represented by a gene. The fitness of each solution (individual) is calculated by comparing the segmentation produced by the solution with the reference segmentation, using five different measures of performance [27] that will be further presented.

As described in the previous section, the segmentation method has a set of five parameters to be optimized:

1. Minimum RMS: the convergence criterion of level set function, defined in terms of the root mean squared (RMS) change in the level set function.

2. *G*_*L*_: Gaussian low factor

3. *G*_*H*_: Gaussian high factor

4. *β*: level set propagation weight

5. *γ*: level set mean curvature weight

Initially a set of solutions (initial population) is generated by computing each parameter value (genes) randomly, within given ranges. As the evolutionary process goes on, the best solutions (fittest individuals) are selected and new solutions (generations) are created from them (reproduction).

The fittest individuals have a larger probability of being selected for reproduction. Furthermore, the best individuals from one generation are kept in the next generation. The evolutionary process stops after a fixed number of generations, and the gene values of the fittest individual are taken as the final (adapted) segmentation parameter values.

#### Fitness Evaluation

**Figure 4:**
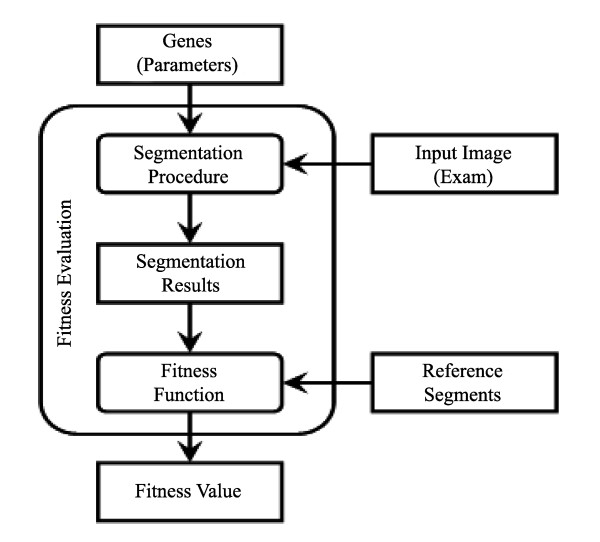
**Fitness evaluation**. The process evaluates a given set of parameters comparing reference data and the result generated by segmentation using the given parameters

The fitness function must indicate the similarity between the segmentation result and the reference segmentation, as shown in figure 4. In mathematical terms, given a set of reference segments *M *and a parameter vector *W *a fitness function *F*(*M,W*) that appropriately expresses the goodness of a segmentation outcome must be defined.

Once the fitness function *F *is chosen, the task of the GA is to search for the parameter vector *W*_*opt*_, for which the value of *F *is minimum:(3)

The fitness function devised in this work is defined as the mean of five scores derived from five disparity metrics proposed by SLiver07 conference [27] that express the level of agreement between the result and the reference surfaces. These metrics try to numerically compare two different segmentations, represented by two different surfaces. In this way, they are measures of spatial differences between the two surfaces. The scores computed over these metrics take into account the uncertainty of a human specialist, in such a way that the score gives the level of agreement between a segmentation outcome and the one produced by a human specialist.

They are, according to [27], and as presented at http://mbi.dkfz-heidelberg.de/grand-challenge2007/sites/eval.htm:

1. "Volumetric overlap (VOE): Is the number of voxels in the intersection of segmentation and reference, divided by the number of voxels in the union of segmentation and reference."

2. "Relative absolute volume difference, in percent (RVD): 1 minus the total volume of the segmentation divided by the total volume of the reference"

3. "Average symmetric absolute surface distance, in millimetres (ASD): the border voxels of segmentation and reference are determined and for each voxel in these sets, the closest voxel in the other set is determined (using Euclidean distance). All these distances are stored, for border voxels from both reference and segmentation. The average of all these distances gives the averages symmetric absolute surface distance."

4. "Symmetric RMS surface distance, in millimetres (RMSSD): is similar to the previous measure, but stores the squared distances between the two sets of border voxels. After averaging the squared values, the root is extracted and gives the symmetric RMS surface distance."

5. "Maximum symmetric absolute surface distance, in millimetres (MSD): is similar to the previous two, but only the maximum of all voxel distances is taken instead of the average."

The scores are calculated based on a number of sample segments manually drawn by specialists for each exam. The reference for each sample is obtained by computing the mean surface among the segmentation produced by all specialists (for details refer to [27]). For each metric the mean of the absolute differences between each specialist result to that reference is computed. These values are taken as the mean human errors. An algorithm with differences exactly equal to these values receives score of 75 points. Segmentation exactly equal to the reference receives the score of 100, and the score decreases linearly as the error increases.

### Nodule and Vessel Segmentation

To segment nodules and vessels we propose a region growing method based on a Gaussian mixture model. Our approach considers three distinct object classes inside the liver: adipose nodules, liver parenchyma and vessels, which may be differentiated by their gray levels. In this work only the adipose nodules, which are less dense than liver parenchyma, are considered.

#### Mixture Model

**Figure 5:**
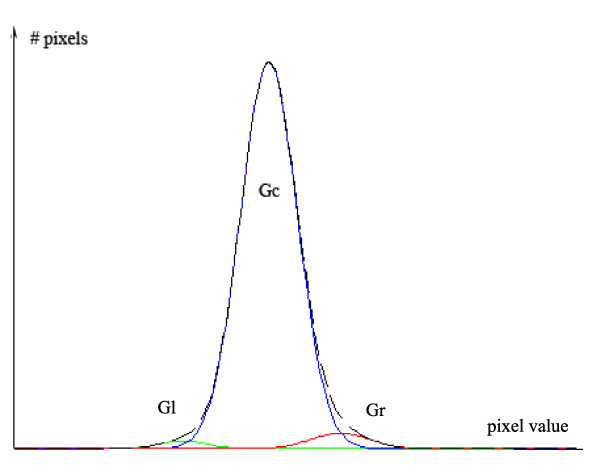
**Gaussian mixture model**. The figure shows the mixture model assumed to segment the liver parenchyma (center gaussian), vessels (right gaussian) and nodules (left gaussian).

The histogram *H*_*i*_(*x*) is computed from the voxels inside the segmented liver volume. The segmentation method uses a mixture model *H*_*i *_*= G*_*l*_*+G*_*c*_*+G*_*r *_of three Gaussians, where *G*_*l *_corresponds to the nodules, *G*_*c *_to liver parenchyma and *G*_*r *_to the vessels (see figure 5):

where *l *is an index for left Gaussian, *c *for centre Gaussian and *r *for right Gaussian; and *K*_*i *_is a constant that multiplies the Gaussian function, with the parameters *μ*_*i *_and *σ*_*i*_.

The algorithm executes 4 steps sequentially:

1. Estimating *G*_*c*_

*G*_*c *_is considered dominant in the mixture model, i.e., *K*_*c *_>> {*K*_*r*_*,K*_*l*_}. Under this assumption it is possible to identify *G*_*c *_using the following procedure.

A new histogram *H*_*c*_(*x*) is computed by taking the minimum value between symmetric points around *x*_*max *_(value where *H*_*i *_reaches its maximum), i.e., *H*_*c*_(*x*) = min [*H*_*i*_(*x*),*H*_*i*_(*2 x*_*max *_-*x*)]. *G*_*c *_is the best fit *H*_*c *_to in the least-squares sense. The non-linear optimizer from Levenberg-Marquadt [24,25] can be used to estimate the parameters *K*_*c*_, *μ*_*c *_and *σ*_*c*_.

2. Estimating *G*_*l*_

The difference *H*^*a*^(*x*) *= H*_*i*_(*x*)*-H*_*c*_(*x*) is computed. That is equivalent to excluding *G*_*c *_from the initial histogram, keeping *G*_*r*_*+G*_*l*_. Next *H*^*a*^(*x*) is set to zero for *x < x*_*max*_. The values *H*_*l*_(*x*) = max [*H*^*a *^(*x*), *H*^*a *^(*2 x*^*a*^_*max *_-*x*)], are then computed, where *x*^*a*^_*max *_is the argument for which *H*^*a*^(*x*) is maximum. This corresponds to taking the maximum value between symmetric points around *x*^*a*^_*max*_. Again, the parameters of *G*_*l *_are estimated using the same non-linear optimizer fitting *G*_*l *_to *H*_*l*_.

3. Estimating *G*_*r*_

*G*_*r *_is estimated from *H*^*b*^(*x*) *= H*_*i*_(*x*)*-H*_*c*_(*x*)*-H*_*l*_(*x*) exactly in the same way as *G*_*l *_was estimated from *H*^*a*^(*x*).

4. Final adjustment

The Levenberg-Marquadt optimizer is applied to find the parameter values that deliver the best fitting of *G*_*l*_*+G*_*c*_*+G*_*r *_to *H*_*i*_.

#### Segmentation of Nodules and Vessels using the mixture model

To segment nodules and vessels a region growing strategy is proposed that bases on the mixture model. The nodules are segmented using the estimated functions *G*_*l *_and *G*_*c *_respectively for the nodules and for the liver parenchyma. Spatial information is used to separate nodules from parenchyma when the intensity is in the range where *G*_*l *_and *G*_*c *_overlap. The region growing approach uses two different thresholds (*T*_*L*_^*lc *^and *T*_*H*_^*lc*^) in such a way that voxels with intensity lower than *T*_*L*_^*lc *^are assigned to nodules, and are used as seeds of a process that aggregates any neighbouring voxel with intensity lower than *T*_*H*_^*lc*^.

**Figure 6:**
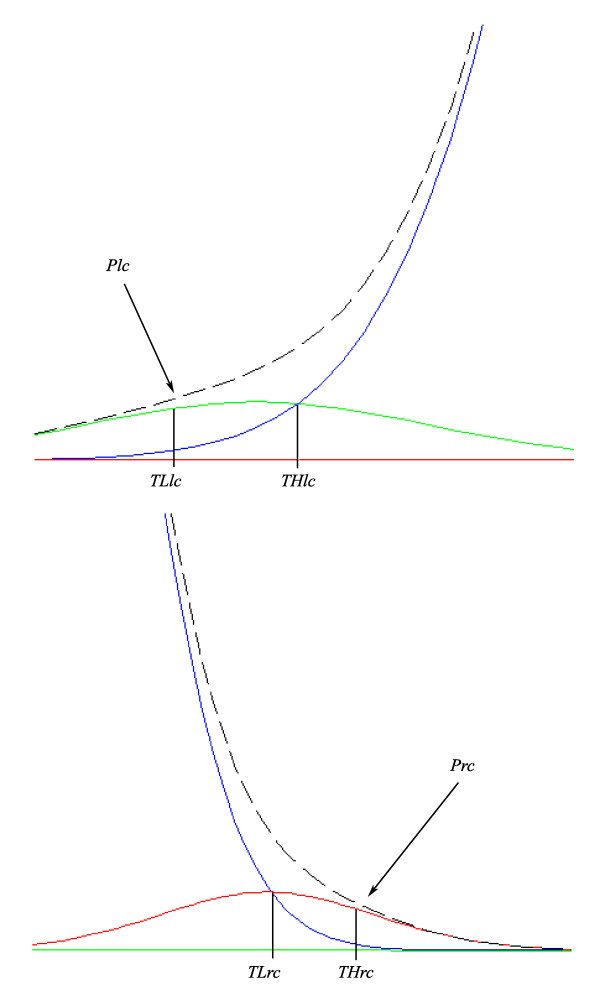
**Thresholds definition**. The thresholds *T*_*L*_^*lc *^and *T*_*H*_^*rc *^are set empirically as the intensity value in which the proportion between voxels belonging to the given *Gaussian *and voxels belonging to the mixture is at least 70%. The thresholds *T*_*L*_^*rc *^and *T*_*H*_^*lc *^are defined as the intensity value where the *Gaussians *intercept.

*T*_*L*_^*lc *^is set empirically as the intensity value in which the proportion *P*^*lc *^between voxels belonging to *G*_*l *_and voxels belonging to the mixture *G*_*l *_*+ G*_*c *_is at least 70%. *T*_*H*_^*lc *^is defined as the intensity value where *G*_*l *_and *G*_*c *_intercept (*T*_*H*_^*lc *^>*T*_*L*_^*lc*^). Figure 6 shows how the thresholds are selected.

The same approach is used to segment vessels. The vessels inside the liver are brighter than liver parenchyma and have intensity distributed according to *G*_*r*_. Again two different thresholds are used to perform a region growing segmentation: *T*_*L*_^*rc *^and *T*_*H*_^*rc*^. The voxels with intensity values greater than *T*_*H*_^*rc *^are classified as vessels straightforwardly and used as seeds for the region growing process that aggregates the neighbouring voxels with intensity value greater than *T*_*L*_^*rc*^.

*T*_*H*_^*rc *^is set empirically as the intensity value in which the proportion *P*^*rc *^of voxels belonging to *G*_*r *_and voxels belonging to the mixture *G*_*r *_*+ G*_*c *_is at least of 70%. *T*_*L*_^*rc *^is set as the intensity value where *G*_*r *_and *G*_*c *_intercept (*T*_*H*_^*rc *^>*T*_*L*_^*rc*^). Figure 6 also illustrates the procedure.

### Identification of Portal and Hepatic veins

Once the vessels are segmented it is necessary to assign them to the hepatic and portal veins, which are the basis for the Couinaud segmentation.

It is assumed that three main branches compose the hepatic veins (which occur in 80% of people). Besides, it is assumed that these branches have predominantly vertical direction, coming from the upper part of the liver to the bottom part.

The hepatic main branches are identified without considering their bifurcations. A vessel is classified as one of the main branches of the hepatic vein, when it is longer than 15% of the liver height. This value was established after testing several different values.

Let *I*_*vessels *_be the binary image containing the vessels mask. The following steps are executed to identify the three main branches of hepatic vein among the segmented vessels:

1. The first slice *k *of *I*_*vessels *_containing vessels is selected.

2. The biggest connected component from the slice *k *is selected and labelled as candidate for hepatic vein main branch.

3. The connected component of the posterior slice with the biggest overlap with the main branch candidate is selected and merged with it.

4. Step 3 is repeated until no more overlapping is observed.

5. If the main branch candidate height is bigger than 15% of the liver height, then this candidate is accepted, otherwise it is discarded, and the process restarts.

6. Steps 1 to 5 are repeated until three main branches have been found.

**Figure 7:**
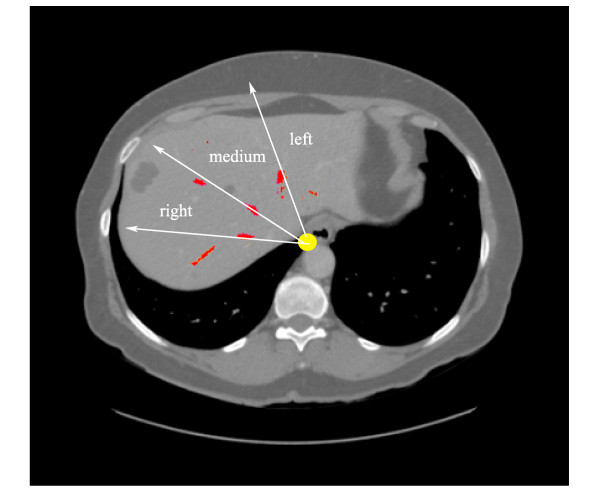
**hepatic vein main branches identification**. The hepatic branches are identified clock wisely: right, medium, and left branches.

The procedure above segments the main branches but doesn't classify them into left, medial or right main branches, as implied by Couinaud anatomical model. To classify the branches a simple method based on a clock is proposed. The centre of the clock is the central axis of the thorax, and the coronal projections of the veins give their classification: they appear in this order clock wisely: right, central, and left. Figure 7 illustrates the procedure.

The portal vein is identified as the biggest connected component among the vessels when the hepatic veins are extracted. The remaining vessels are merged to the hepatic branches they are connected to, or classified as auxiliary vessels, when they don't.

### Couinaud Liver Segmentation

The division of the liver into Couinaud segments is based on liver anatomy. The geometric approach proposed in this work creates four planes: three approximately vertical, following the orientation of each hepatic vein main branch and one approximately horizontal, following the orientation of portal vein.

To determine each of these planes a simple algorithm of geometric fit, based on least squares method, is proposed. The voxels belonging to each branch of hepatic vein and to the portal vein are selected and a specific plane with the characteristics of the model (near vertical or horizontal) is computed. The four computed planes determine the limit of the 8 Couinaud regions.

## Results and Discussion

### Liver Segmentation

In order to evaluate the performance of the proposed method, a software prototype was developed in C++/C#. The prototype uses the ITK library [28] which implements the level set framework used in the segmentation method. It is also possible to visualize the results in different slice directions, as well as the 3D organ models. An additional movie showing the prototype is available in http://www.lvc.ele.puc-rio.br/projects/Liver3D/3dliver.avi.

It was also developed an environment where the user can specify different GA configurations, enable and disable search of parameters, and define their default values and search ranges. It is possible to define the training set, and fitness aggregation type (mean of fitness values, or minimum fitness value).

An evaluation of the optimal GA configuration values for the optimization of the segmentation parameters goes beyond the scope of this paper, so the GA was configured experimentally, and its convergence was verified visually. The GA configuration values are the following:

• Number of generations = 30;

• Population Size = 30;

• Initial crossover rate = 0.8;

• Final crossover rate = 0.65;

• Initial mutation rate = 0.1;

• Final mutation rate = 0.8;

• Initial Steady State rate = 0.8;

• Final Steady State rate = 0.2;

• Number of sequenced experiments = 2;

• Rate of seed from the first experiment to the second = 0.1;

In our experiment a set of five exams was used for estimation of segmentation parameter values. The method was evaluated upon the other 15 exams available on the dataset. Both input and reference data were provided by SLiver07 conference [27]. The parameter values found by the GA in our experiment were:

1. Minimum RMS: 0.0209

2. *G*_*L*_: 0.3859

3. *G*_*H*_: 0.2809

4. *β*: -5.1929

5. *γ*: 217.414

The results obtained by the presented approach are shown in Table 1, where both the metric value and its corresponding score are shown in each cell for the different evaluation metrics. We compiled our results, showing the best, worst, and mean results for all test set. It is possible to notice that some metrics such as RMSSD and MSD are more rigorous than other, but the algorithm achieved good results. Recall that, according to [27], the maximum score is equal to 100 and corresponds to a perfect match with the reference. A score equal to 75 corresponds roughly to a segmentation done manually by a specialist. For scores higher than 60 disparities from the reference segmentation are hardly detectable visually.

**Table 1:** Liver segmentation results

Evaluation	Best	Worst	Mean
VOE	5.45	12.07	7.35
(Score)	(78.70)	(52.82)	(71.29)

RVD	-0.63	8.12	-2.19
(Score)	(96.63)	(56.80)	(82.27)

ASD	0.76	3.57	1.35
(Score)	(80.85)	(10.70)	(66.25)

RMSSD	1.69	8.22	3.05
(Score)	(76.46)	(0)	(58.58)

MSD	17.03	55.09	26.81
(Score)	(77.59)	(27.21)	(64.72)

Total Score	82.05	29.57	68.62

The table shows the results for liver segmentation using five different metrics considering the best, worst and mean outcomes.

**Figure 8:**
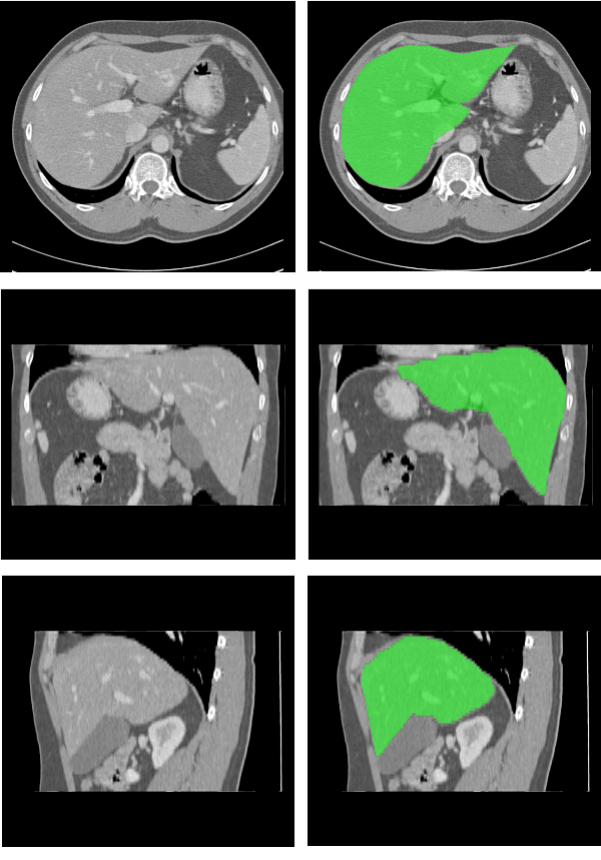
**Best result obtained: axial view; coronal view; sagittal view**. The liver segmentation result appears in green.

The method attained a good performance in 17 of the 20 exams, in which the overall score was above 65. Only small errors were detected within these exams. Figure 8 shows the result obtained in the best result, with overall score 82.05. Notice that the liver boundaries are accurately defined.

**Figure 9:**
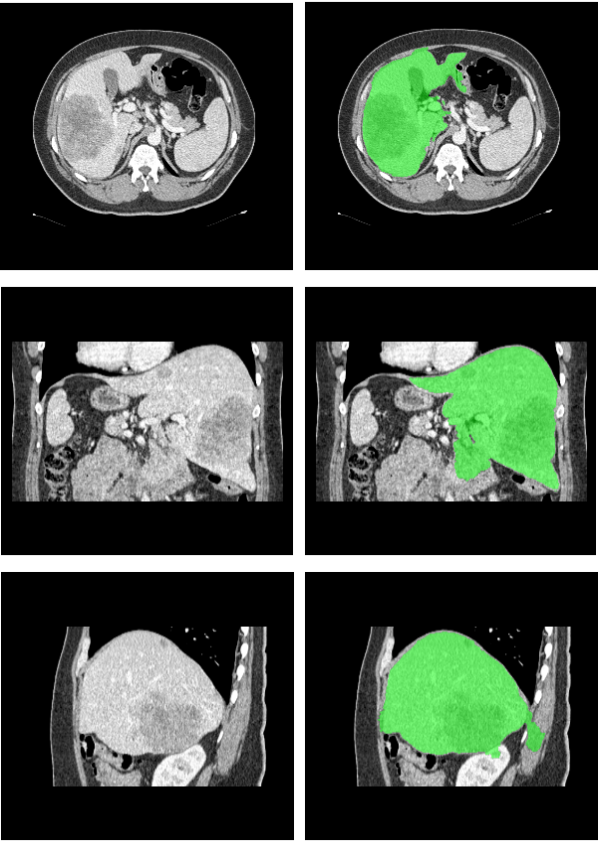
**Leak caused by a big nodule: axial view; coronal view; sagittal view**. The liver segmentation result appears in green.

In 3 exams, though, the results contain some significant errors that can be detected visually. The exam with the lowest score among all tested exams is depicted in Figure 9, with an overall score of 29.57. In this exam the liver has a large nodule, which causes a leak of the segmented region towards adjacent darker structures. This can be easily explained by the adopted heuristic that fits a Gaussian to characterize the liver parenchyma. When a nodule with size comparable to the liver parenchyma is present, this approach is susceptible to imperfections, as the histogram will not be Gaussian shaped. Yet, the final contour defines properly most part of the liver. This robust behavior should be credited to the way *T*_*L *_and *T*_*H *_are computed, which is performed at each slice, having their values adapted to different regions of the liver.

The result of another exam with low score (46.18) has a single major error, caused by a peripheral nodule not classified as liver. This is again due to the adopted heuristic that models the liver tissue by a single Gaussian. As the nodule in this exam appears much darker than the liver parenchyma, its voxel intensities lie outside the range [*T*_*L*_*,T*_*H*_] defined by the Gaussian fit. As the nodule is at the periphery, the morphological hole filling filter was not able to correct the error, and therefore the nodule region was not included in the final result. Important metrics such as RMSSD and MSD, which take into account the maximum differences between the reference and the resulting surfaces, generated low scores.

In the last exam with poor performance, we see a leak towards the mediastinum, with overall score of 45.58. This leak towards the mediastinum is commonly observed in many different approaches, mainly because the mediastinum share some intensity values with the liver and is anatomically close to the liver in a wide extent. This makes it hard to define properly the boundary between them in some cases. Some post processing can be done to avoid it, as the mediastinum has a known relative position to the liver. Another possibility would be the inclusion of the advection term to create a barrier between the liver and the mediastinum. The operator using a proper user interface could manually define this.

Our results can be compared with the results of other approaches tested upon the data and metrics compliant with the liver segmentation competition held in the Sliver07 conference. If compared with other automatic and semi-automatic methods presented in the conference's website, all of them using the same dataset used in this work, our method had a good performance, being ranked as the 6th best method among 26 (2008).

The estimation of parameters may take a long time (about one week), but it has to be executed just once, and the values are used for all exams from then on. Once the parameters have been estimated, the method is quite fast. The computation time depends on the exam size: a 210 slices exam, takes 204 seconds to segment the liver, while a 110 slices exam takes 105 seconds, on a AMD Athlon 3.0 GHz with 2GB RAM.

#### Analysis of sensibility

**Figure 10:**
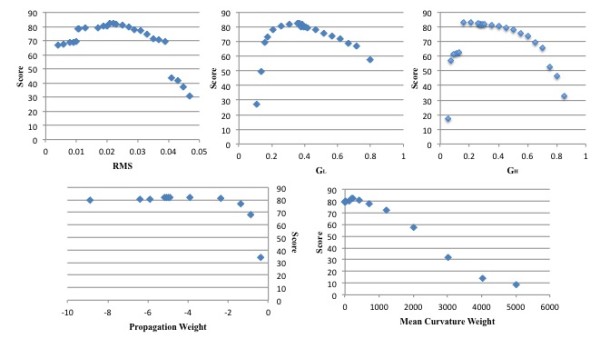
**Analysis of parameters sensibility: RMS, G**_**L**_**, G**_**H**_**, propagation weight, and mean curvature weight**. The charts show the range of values and their performance result for each parameter.

An experiment for analysis of parameters sensibility was conducted involving the exam in which we obtained the best result and the optimal values. Each parameter was varied individually until the performance decreased consistently, in order to assess the range of values for which they work satisfactorily. Figure 10 shows the results for each parameter.

It is possible to see that Minimum RMS values range from 0.01 to 0.03 without losing much performance. G_L _and G_H _range from 0.3 to 0.5 and from 0.2 to 0.5 respectively with good performances. The mean curvature weight ranges satisfactorily from near 0 to 1000. It is interesting to observe the behaviour of propagation weight; it seems to have a constant asymptotic behaviour when getting far from zero value (we tested with values of the order of tens of thousands). A high propagation weight value would probably affect the fine-tuning of level sets near to the liver border, but since our propagation image values are very low there, it seems not to have a great impact on performance.

### Nodules and Vessels Segmentation

**Figure 11:**
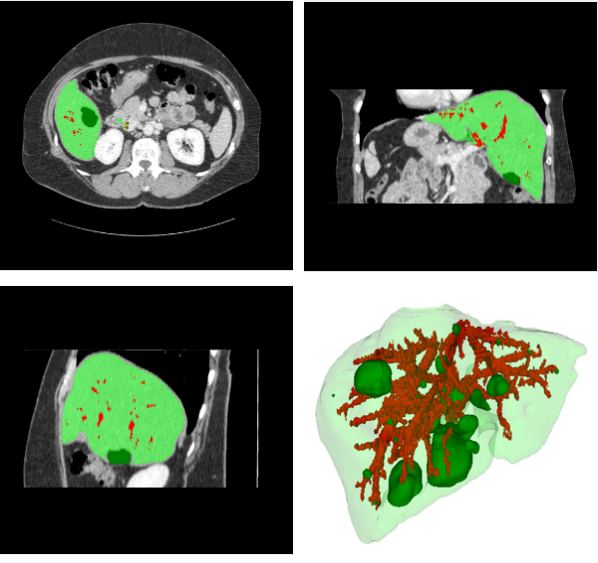
**Vessels and nodules segmentation: axial, coronal, sagittal and 3D views**. The liver appears in light green, the vessels in red and the nodules in dark green.

The evaluation of nodules and vessels segmentation was visual, since no reference data is available. Figure 11 shows examples of the outcome. The results are visually consistent, with brighter regions classified as vessels, and darker as nodules.

### Identification of Portal and Hepatic veins

**Figure 12:**
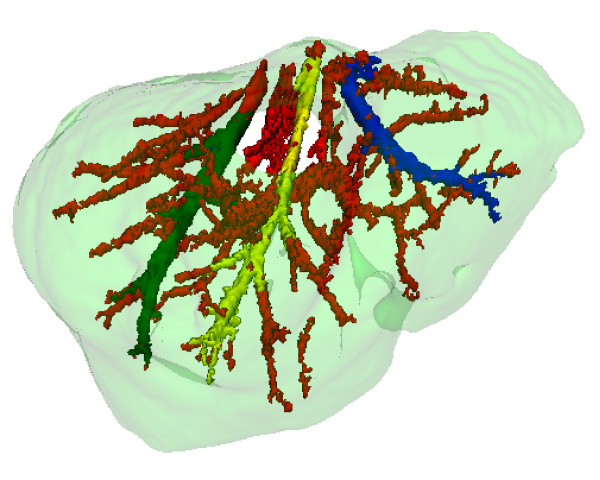
**Hepatic vein main branches identification**. The right main branch appears in green, the medium in yellow, and the left in blue. The other vessels appear in red.

**Figure 13:**
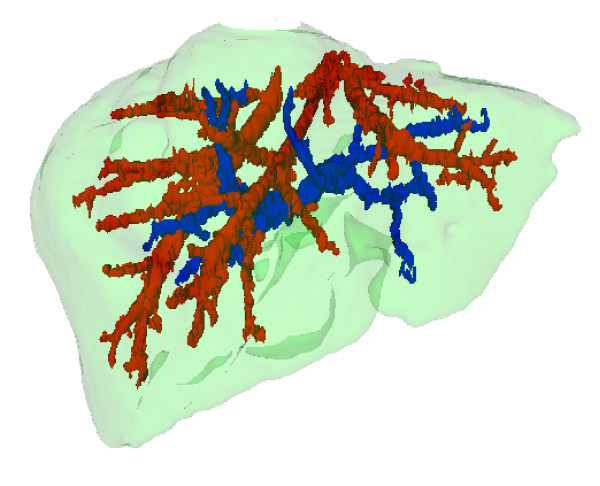
**Hepatic and Portal veins segmentation**. The hepatic veins appear in red and the portal vein in blue.

The evaluation of the identification of portal and hepatic veins was also visual. This evaluation is less subjective, since a simple check of the result with the anatomical model reduces the possibilities of evaluation mistakes. Figure 12 shows an example where the main branches of the hepatic vein appear in green, yellow and blue. Using these main branches, the hepatic vein and the portal vein are identified, as shown respectively in red and blue in figure 13. A specialist considered the results obtained consistent with liver anatomy.

### Couinaud Liver segmentation

**Figure 14:**
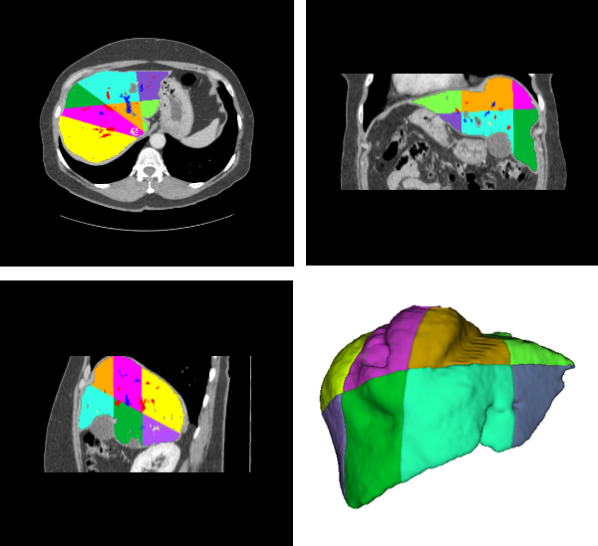
**Couinaud segmentation: axial, coronal, sagittal and 3d views**. The Couinaud segments appear in different colors, following the model described in figure 1.

The evaluation of the liver division into Couinaud segments was again visual. This evaluation is substantially subjective, and even trained doctors may present results slightly different. A specialist was asked to evaluate the results, and although the results are consistent with the adopted anatomic model, some inconsistencies with other anatomic models present in the literature were found. Figure 14 shows the obtained results.

## Conclusions

We have presented a methodology to segment the liver and its internal structures. We also propose a method to separate the liver into segments according to the Couinaud anatomical model.

Firstly the liver was segmented using a method based on level sets and an evolutionary method to estimate its parameters. The use of level sets led to a good accuracy in the tested exams, and the evolutionary approach based on GAs to estimate segmentation parameters, has been shown robust in our experiments. The GA based method for model adaptation managed to capture the inherent subjectivity present in the visual segmentation of organs in computer tomography.

The method for nodules and vessels segmentation performed well for most CT exams of the available dataset. Yet, some limitations became apparent, especially when there are nodules or veins close to the liver periphery, and also in the presence of nodules with volume similar to the liver parenchyma. These cases indicate directions for the continuation of this research aiming at a further refinement of the proposed method. The identification of portal and hepatic veins also generated results consistent anatomically.

Finally, the geometric approach to fit planes to the hepatic and portal veins managed to define Couinaud regions. The quality assessment was also visual, and the specialist consulted to evaluate the results found some inconsistencies with other anatomic models present in the literature, though the results were consistent with the Couinaud model. This result brings up a discussion about the convergence of the different existing theoretical anatomic models for the liver [29-31].

The authors believe that the method to segment the liver may be applied with some adjustments to segment other organs, especially when the distribution of voxel intensities is nearly Gaussian shaped. The evolutionary approach can be also used to estimate segmentation parameters for other organs, if a proper training set can be provided.

## Competing interests

The authors declare that they have no competing interests.

## Authors' contributions

DABO and RQF conceived the theoretical model to segment the liver and its internal structures. MMC defined the anatomical model, giving the medicine basis for the ideas implemented. DABO implemented the methodology, including segmentation of liver, nodules, vessels and identification of hepatic and portal veins, and Couinaud segmentation. MMC evaluated the results generated. All authors read and approved the final manuscript.
